# 5,6-Dimethyl-1,2,4-triazin-3-amine

**DOI:** 10.1107/S1600536811051920

**Published:** 2011-12-07

**Authors:** Man-Hua Wu, Qi-Ming Qiu, Sen Gao, Qiong-Hua Jin, Cun-Lin Zhang

**Affiliations:** a Department of Chemistry, Capital Normal University, Beijing 100048, People’s Republic of China; b Key Laboratory of Terahertz Optoelectronic, Ministry of Education, Department of Physics, Capital Normal University, Beijing 100048, People’s Republic of China

## Abstract

In the crystal structure of the title compound, C_5_H_8_N_4_, adjacent mol­ecules are connected through N—H⋯N hydrogen bonds, resulting in a zigzag chain along [100]. The amino groups and heterocyclic N atoms are involved in further N—H⋯N hydrogen bonds, forming *R*_2_^2^(8) motifs.

## Related literature

For the biological and medical applications of triazine, see: Anderson *et al.*(2003 ▶); Gavai *et al.* (2009 ▶); Hunt *et al.* (2004 ▶). For the structures of complexes containing triazine, see: Drew *et al.* (2001 ▶); Li *et al.* (2009 ▶); Machura *et al.* (2008 ▶). For the structures of complexes containing the title compound, see: Jiang *et al.* (2011 ▶); Self *et al.* (1991 ▶); Wu *et al.* (2011 ▶). For the structures of compounds containing 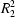
(8)-type hydrogen bonds, see: Etter (1990 ▶); Glidewell *et al.* (2003 ▶).
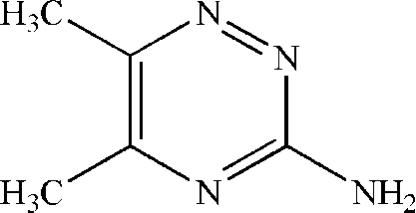


## Experimental

### 

#### Crystal data

C_5_H_8_N_4_
*M* *_r_* = 124.14
Orthorhombic, 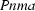

*a* = 7.4877 (8) Å
*b* = 6.7530 (7) Å
*c* = 12.6615 (13) Å
*V* = 640.22 (12) Å^3^
*Z* = 4
Mo *K*α radiation
μ = 0.08 mm^−1^
*T* = 293 K
0.50 × 0.39 × 0.38 mm

#### Data collection

Bruker SMART CCD area-detector diffractometer
Absorption correction: multi-scan (*SADABS*; Bruker, 2007 ▶) *T*_min_ = 0.960, *T*_max_ = 0.969
2997 measured reflections
614 independent reflections
421 reflections with *I* > 2σ(*I*)
*R*_int_ = 0.034

#### Refinement

*R*[*F* ^2^ > 2σ(*F* ^2^)] = 0.048
*wR*(*F* ^2^) = 0.157
*S* = 1.11
614 reflections
58 parameters
H-atom parameters constrained
Δρ_max_ = 0.26 e Å^−3^
Δρ_min_ = −0.16 e Å^−3^

### 

Data collection: *SMART* (Bruker, 2007 ▶); cell refinement: *SAINT-Plus* (Bruker, 2007 ▶); data reduction: *SAINT-Plus*; program(s) used to solve structure: *SHELXS97* (Sheldrick, 2008 ▶); program(s) used to refine structure: *SHELXL97* (Sheldrick, 2008 ▶); molecular graphics: *SHELXTL* (Sheldrick, 2008 ▶); software used to prepare material for publication: *SHELXTL*.

## Supplementary Material

Crystal structure: contains datablock(s) global, I. DOI: 10.1107/S1600536811051920/rn2097sup1.cif

Structure factors: contains datablock(s) I. DOI: 10.1107/S1600536811051920/rn2097Isup2.hkl

Supplementary material file. DOI: 10.1107/S1600536811051920/rn2097Isup3.cml

Additional supplementary materials:  crystallographic information; 3D view; checkCIF report

## Figures and Tables

**Table 1:** Hydrogen-bond geometry (Å, °)

*D*—H⋯*A*	*D*—H	H⋯*A*	*D*⋯*A*	*D*—H⋯*A*
N4—H4*A*⋯N3^i^	0.86	2.19	3.045 (4)	179
N4—H4*B*⋯N2^ii^	0.86	2.09	2.947 (4)	176

Symmetry codes: (i) 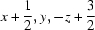; (ii) 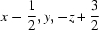.

**Fig. 1.**
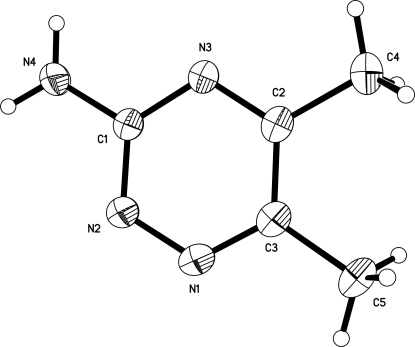
Molecular structure of (I) with displacement ellipsoids drawn at the 50% probability level.

**Fig. 2.**
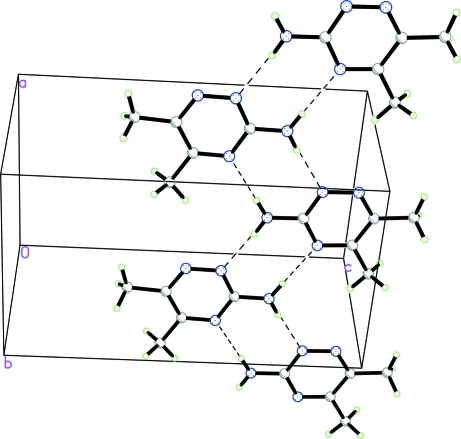
Crystal packing for (I) with hydrogen bonds shown as dashed lines.

## supplementary crystallographic information

### Comment

The heterocyclic nitrogen compounds containing 1,2,4-triazine moieties have drawn much attention in recent years, owing to their interesting biological and medicinal properties (Anderson *et al.*, 2003; Gavai *et al.*, 2009; Hunt *et al.*,2004). They usually act as efficient ligands in supramolecular compounds (Drew *et al.*, 2001; Li *et al.*, 2009; Machura *et al.*, 2008). The title compound (I) has been used as a multidentate ligand to form poly-nuclear complexes (Self *et al.*, 1991). In (I), hydrogen bonds are formed between the NH groups of amino group and the N atoms.

We are interested in synthesizing new transition metal complexes containing (I) (Jiang *et al.*, 2011; Wu *et al.*, 2011). The title compound was unexpectedly obtained in the course of synthesizing Cu(I) complexes.

In the title compound, adjacent molecules are connected by intermolecular N—H···N hydrogen bonds to form a zigzag structure (Fig. 2). In the crystal structure, the amino groups and heterocyclic N atoms are involved in hydrogen bonds,forming *R*_2_^2^(8) type hydrogen bonds (Etter, 1990; Glidewell *et al.*, 2003).

### Experimental

A mixture of CuCN and ADMT (ADMT=3-amino-5,6-dimethyl- 1,2,4-triazine) in molar ratio of 1:1 in the mixed solution of CH_3_CN (7 ml)/ CH_3_OH (3 ml) was stirred for 3 h,then filtered. Pale yellow crystals were obtained from the filtrate after standing at room temperature for several days.

### Refinement

The final refinements were performed with isotropic thermal parameters. All hydrogen atoms were located in the calculated sites and included in the final refinement in the riding model approximation with displacement parameters derived from the parent atoms to which they were bonded. The ratios of H atom *U*_iso_ to C atom *U*_eq_ are 1.5. The ratios of H atom *U*_iso_ to N atom *U*_eq_ are 1.2.

### Crystal data

**Table:**

C_5_H_8_N_4_	*D*_x_ = 1.278 Mg m^−^^3^
*M**_r_* = 124.14	Mo *K*α radiation, λ = 0.71073 Å
Orthorhombic, *P**n**m**a*	Cell parameters from 1029 reflections
*a* = 7.4877 (8) Å	θ = 2.7–28.0°
*b* = 6.7530 (7) Å	µ = 0.08 mm^−^^1^
*c* = 12.6615 (13) Å	*T* = 293 K
*V* = 640.22 (12) Å^3^	Block, yellow
*Z* = 4	0.50 × 0.39 × 0.38 mm
*F*(000) = 264	

### Data collection

**Table:**

Bruker SMART CCD area-detector diffractometer	614 independent reflections
Radiation source: fine-focus sealed tube	421 reflections with *I* > 2σ(*I*)
graphite	*R*_int_ = 0.034
phi and ω scans	θ_max_ = 25.0°, θ_min_ = 3.2°
Absorption correction: multi-scan (*SADABS*; Bruker, 2007)	*h* = −7→8
*T*_min_ = 0.960, *T*_max_ = 0.969	*k* = −8→7
2997 measured reflections	*l* = −14→15

### Refinement

**Table:**

Refinement on *F*^2^	Primary atom site location: structure-invariant direct methods
Least-squares matrix: full	Secondary atom site location: difference Fourier map
*R*[*F*^2^ > 2σ(*F*^2^)] = 0.048	Hydrogen site location: inferred from neighbouring sites
*wR*(*F*^2^) = 0.157	H-atom parameters constrained
*S* = 1.11	*w* = 1/[σ^2^(*F*_o_^2^) + (0.0627*P*)^2^ + 0.3625*P*] where *P* = (*F*_o_^2^ + 2*F*_c_^2^)/3
614 reflections	(Δ/σ)_max_ < 0.001
58 parameters	Δρ_max_ = 0.26 e Å^−^^3^
0 restraints	Δρ_min_ = −0.16 e Å^−^^3^

### Special details

**Table:**

Geometry. All e.s.d.'s (except the e.s.d. in the dihedral angle between two l.s. planes) are estimated using the full covariance matrix. The cell e.s.d.'s are taken into account individually in the estimation of e.s.d.'s in distances, angles and torsion angles; correlations between e.s.d.'s in cell parameters are only used when they are defined by crystal symmetry. An approximate (isotropic) treatment of cell e.s.d.'s is used for estimating e.s.d.'s involving l.s. planes.
Refinement. Refinement of *F*^2^ against ALL reflections. The weighted *R*-factor *wR* and goodness of fit *S* are based on *F*^2^, conventional *R*-factors *R* are based on *F*, with *F* set to zero for negative *F*^2^. The threshold expression of *F*^2^ > σ(*F*^2^) is used only for calculating *R*-factors(gt) *etc*. and is not relevant to the choice of reflections for refinement. *R*-factors based on *F*^2^ are statistically about twice as large as those based on *F*, and *R*- factors based on ALL data will be even larger.

### Fractional atomic coordinates and isotropic or equivalent isotropic displacement parameters (Å^2^)

**Table:**

	*x*	*y*	*z*	*U*_iso_*/*U*_eq_	Occ. (<1)
N1	1.0561 (4)	0.2500	0.5062 (2)	0.0514 (9)	
N2	1.0506 (4)	0.2500	0.6123 (2)	0.0499 (8)	
N3	0.7314 (4)	0.2500	0.60746 (19)	0.0466 (8)	
N4	0.8858 (4)	0.2500	0.7657 (2)	0.0609 (10)	
H4A	0.9838	0.2500	0.8011	0.073*	
H4B	0.7850	0.2500	0.7982	0.073*	
C1	0.8903 (4)	0.2500	0.6589 (2)	0.0447 (9)	
C2	0.7407 (5)	0.2500	0.5030 (2)	0.0469 (9)	
C3	0.9072 (5)	0.2500	0.4511 (2)	0.0477 (9)	
C4	0.5682 (5)	0.2500	0.4422 (3)	0.0678 (12)	
H4C	0.5720	0.3512	0.3890	0.102*	0.50
H4D	0.4708	0.2755	0.4896	0.102*	0.50
H4E	0.5516	0.1233	0.4093	0.102*	0.50
C5	0.9233 (5)	0.2500	0.3332 (2)	0.0624 (11)	
H5A	0.8506	0.1461	0.3044	0.094*	0.50
H5B	1.0456	0.2286	0.3137	0.094*	0.50
H5C	0.8839	0.3753	0.3060	0.094*	0.50

### Atomic displacement parameters (Å^2^)

**Table:**

	*U*^11^	*U*^22^	*U*^33^	*U*^12^	*U*^13^	*U*^23^
N1	0.0485 (19)	0.063 (2)	0.0425 (16)	0.000	0.0087 (13)	0.000
N2	0.0412 (17)	0.069 (2)	0.0395 (16)	0.000	0.0016 (12)	0.000
N3	0.0431 (16)	0.062 (2)	0.0346 (15)	0.000	−0.0012 (11)	0.000
N4	0.0382 (16)	0.105 (3)	0.0391 (16)	0.000	−0.0064 (12)	0.000
C1	0.0417 (19)	0.057 (2)	0.0350 (17)	0.000	−0.0008 (13)	0.000
C2	0.054 (2)	0.050 (2)	0.0374 (19)	0.000	−0.0014 (14)	0.000
C3	0.055 (2)	0.049 (2)	0.0397 (19)	0.000	0.0031 (16)	0.000
C4	0.060 (2)	0.097 (3)	0.047 (2)	0.000	−0.0118 (17)	0.000
C5	0.075 (3)	0.074 (3)	0.0376 (19)	0.000	0.0075 (18)	0.000

### Geometric parameters (Å, °)

**Table:**

N1—C3	1.315 (4)	C2—C4	1.503 (5)
N1—N2	1.344 (4)	C3—C5	1.498 (4)
N2—C1	1.338 (4)	C4—H4C	0.9600
N3—C2	1.325 (4)	C4—H4D	0.9600
N3—C1	1.356 (4)	C4—H4E	0.9600
N4—C1	1.353 (4)	C5—H5A	0.9600
N4—H4A	0.8600	C5—H5B	0.9600
N4—H4B	0.8600	C5—H5C	0.9600
C2—C3	1.409 (5)		
			
C3—N1—N2	120.2 (3)	C2—C3—C5	122.4 (3)
C1—N2—N1	117.9 (3)	C2—C4—H4C	109.5
C2—N3—C1	115.7 (3)	C2—C4—H4D	109.5
C1—N4—H4A	120.0	H4C—C4—H4D	109.5
C1—N4—H4B	120.0	C2—C4—H4E	109.5
H4A—N4—H4B	120.0	H4C—C4—H4E	109.5
N2—C1—N4	117.6 (3)	H4D—C4—H4E	109.5
N2—C1—N3	125.2 (3)	C3—C5—H5A	109.5
N4—C1—N3	117.3 (3)	C3—C5—H5B	109.5
N3—C2—C3	120.8 (3)	H5A—C5—H5B	109.5
N3—C2—C4	117.7 (3)	C3—C5—H5C	109.5
C3—C2—C4	121.5 (3)	H5A—C5—H5C	109.5
N1—C3—C2	120.2 (3)	H5B—C5—H5C	109.5
N1—C3—C5	117.4 (3)		
			
C3—N1—N2—C1	0.0	N2—N1—C3—C2	0.0
N1—N2—C1—N4	180.0	N2—N1—C3—C5	180.0
N1—N2—C1—N3	0.000 (1)	N3—C2—C3—N1	0.0
C2—N3—C1—N2	0.000 (1)	C4—C2—C3—N1	180.0
C2—N3—C1—N4	180.0	N3—C2—C3—C5	180.0
C1—N3—C2—C3	0.0	C4—C2—C3—C5	0.0
C1—N3—C2—C4	180.0		

### Hydrogen-bond geometry (Å, °)

**Table:**

*D*—H···*A*	*D*—H	H···*A*	*D*···*A*	*D*—H···*A*
N4—H4A···N3^i^	0.86	2.19	3.045 (4)	179.
N4—H4B···N2^ii^	0.86	2.09	2.947 (4)	176.

Symmetry codes: (i) *x*+1/2, *y*, −*z*+3/2; (ii) *x*−1/2, *y*, −*z*+3/2.
